# 4-(Benzyl­ideneamino)benzene­sulfonamide

**DOI:** 10.1107/S1600536809030256

**Published:** 2009-08-08

**Authors:** Bradley T. Loughrey, Michael L. Williams, Peter C. Healy

**Affiliations:** a Eskitis Institute for Cell and Molecular Therapies, Griffith University, Brisbane 4111, Australia

## Abstract

The title compound, C_13_H_12_N_2_O_2_S, formed by Schiff base condensation of benzaldehyde with sulfanilamide, crystallizes as discrete mol­ecular species linked by N—H⋯N and N—H⋯O hydrogen bonds between the sulfamide nitro­gen H atoms and the aza­methine N and one sulfamide O atom, respectively, forming a two-dimensional array in the *bc* plane. The aza­methine group is rotated slightly out of the benzaldehyde benzene plane [C—C—C—N torsion angle = 8.1 (3)°], while the dihedral angle between the two benzene rings is 30.0 (1)°.

## Related literature

Condensation of substituted benzaldehydes with sulfanilamide yields a diverse array of Schiff bases which display inter­esting enzymatic inhibition, see Supuran *et al.* (1996 ▶); Lin *et al.* (2008 ▶). For our ongoing studies on the synthesis, structures and biological activity of organometallic Cp*Ru(II) arene complexes Loughrey *et al.* (2008 ▶, 2009 ▶). For related structures, see Chumakov *et al.* (2006 ▶); Subashini *et al.* (2009 ▶).
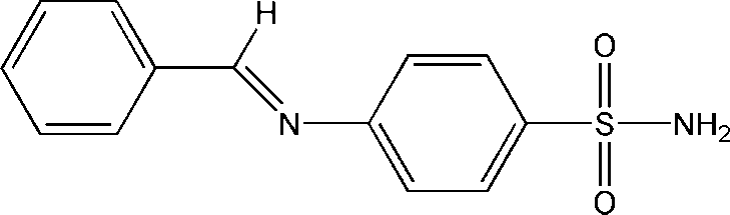


## Experimental

### 

#### Crystal data

C_13_H_12_N_2_O_2_S
*M* *_r_* = 260.32
Monoclinic, 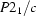

*a* = 14.5206 (8) Å
*b* = 11.4992 (6) Å
*c* = 7.7846 (5) Å
β = 103.287 (6)°
*V* = 1265.04 (13) Å^3^
*Z* = 4
Mo *K*α radiation
μ = 0.25 mm^−1^
*T* = 296 K
0.43 × 0.31 × 0.20 mm

#### Data collection

Oxford-Diffraction Gemini S Ultra diffractometer
Absorption correction: multi-scan (*CrysAlis RED*; Oxford Diffraction, 2007 ▶) *T*_min_ = 0.900, *T*_max_ = 0.952
8991 measured reflections
2253 independent reflections
1928 reflections with *I* > 2σ(*I*)
*R*_int_ = 0.018

#### Refinement

*R*[*F* ^2^ > 2σ(*F* ^2^)] = 0.031
*wR*(*F* ^2^) = 0.085
*S* = 1.05
2253 reflections
163 parameters
H-atom parameters constrained
Δρ_max_ = 0.27 e Å^−3^
Δρ_min_ = −0.24 e Å^−3^

### 

Data collection: *CrysAlis CCD* (Oxford Diffraction, 2007 ▶); cell refinement: *CrysAlis RED* (Oxford Diffraction, 2007 ▶); data reduction: *CrysAlis RED*; program(s) used to solve structure: *SIR97* (Altomare *et al.*, 1999 ▶); program(s) used to refine structure: *SHELXL97* (Sheldrick, 2008 ▶); molecular graphics: *ORTEP-3 for Windows* (Farrugia, 1997 ▶); software used to prepare material for publication: *PLATON* (Spek, 2009 ▶).

## Supplementary Material

Crystal structure: contains datablocks global, I. DOI: 10.1107/S1600536809030256/tk2519sup1.cif

Structure factors: contains datablocks I. DOI: 10.1107/S1600536809030256/tk2519Isup2.hkl

Additional supplementary materials:  crystallographic information; 3D view; checkCIF report

## Figures and Tables

**Table 1:** Hydrogen-bond geometry (Å, °)

*D*—H⋯*A*	*D*—H	H⋯*A*	*D*⋯*A*	*D*—H⋯*A*
N1—H11⋯N4^i^	0.86	2.14	2.9955 (18)	171
N1—H12⋯O11^ii^	0.87	2.13	2.9845 (19)	171

Symmetry codes: (i) 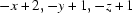; (ii) 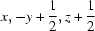.

**Fig. 1.**
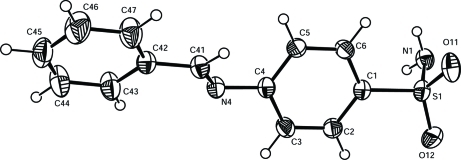
The structure of (I), with atom labels and 40% probability displacement ellipsoids for the non-H atoms.

**Fig. 2.**
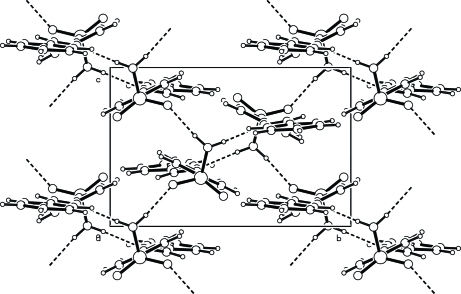
Intermolecular hydrogen bonding interactions (dashed lines) for (I) leading a 2D array in the bc plane, viewed down the a axis.

## supplementary crystallographic information

### Comment

Condensation of substituted benzaldehydes with sulfanilamide yields a diverse array of Schiff bases which display interesting enzymatic inhibition towards the carbonic anhydrase (CA) isozymes CA I, II and IV (Supuran *et al.*, 1996) and the *cyclo*-oxogenase (COX) enzymes COX-1 and COX-2 (Lin *et al.*, 2008). As part of our ongoing studies on the synthesis, structures and biological activity of organometallic Cp*Ru(II) arene complexes with these and related benzenesulfonamides [Cp*Ru(R—Ph—SO_2_NH_2_)]*X* (Loughrey *et al.*, 2008, 2009) we have prepared and determined the crystal structure of the title compound (I).

The crystal structure of (I) consists of discrete molecules (Fig. 1) with bond lengths in the normal range expected for this class of compound (Chumakov *et al.*, 2006; Subashini *et al.*, 2009). The –CH=N– azomethine group is rotated slightly out of the plane of the benzaldehyde benzene ring with the torsion angle C43—C42—C41—N4 = 8.1 (3)°. The dihedral angle between the two benzene rings is 30.0 (1)°. In the crystal lattice, the sulfamide nitrogen protons form N—H···N and N—H···O intermolecular hydrogen bonds with the azamethine nitrogen and the sulfamide oxygen O11 (Table 1, Fig. 2).

### Experimental

Compound (I) was prepared according to established procedures (Lin *et al.*, 2008). Sulfanilamide (1.0 g, 5.81 mmol) was dissolved in a minimum quantity of ethanol and the resulting solution heated to reflux. Benzaldehyde (0.59 ml, 5.81 mmol) was added dropwise over a period of 5 minutes, during which time a fine white precipitate started to form. The mixture was heated at reflux for a further 3 h, after which the solvent was cooled and concentrated *in vacuo*. The resulting white, crystalline precipitate was filtered and washed with cold ethanol. Yield = 1.47 g, 97%. *M*.p. 462–465 K. NMR ^1^H (d_6_ DMSO), δ 7.35 (br s, 2H, NH_2_), 7.37 - 7.40 (m, 2H, C_6_H_4_*ortho*), 7.51 - 7.57 (m, 3H, C_6_H_5_*meta*, *para*), 7.84 - 7.87 (m, 2H, C_6_H_4_*meta*), 7.94 - 7.97 (m, 2H, C_6_H_5_*ortho*), 8.64 (s, 1H, CH=N). Crystals suitable for X-ray diffraction studies were grown by slow evaporation of an acetone solution of (I).

### Refinement

H atoms attached to carbon were constrained as riding atoms with C–H set to 0.95 Å, and with *U*_iso_(H) values set to 1.2*U*_eq_ of the parent atom. The N protons were located in Fourier difference maps and constrained as riding atoms with N–H set to 0.86 - 0.87 Å, and with *U*_iso_(H) values set to 1.2*U*_eq_ of the parent atom.

### Crystal data

**Table:**

C_13_H_12_N_2_O_2_S	*F*(000) = 544
*M**_r_* = 260.32	*D*_x_ = 1.367 Mg m^−^^3^
Monoclinic, *P*2_1_/*c*	Mo *K*α radiation, λ = 0.71070 Å
Hall symbol: -P 2ybc	Cell parameters from 6065 reflections
*a* = 14.5206 (8) Å	θ = 3.2–32.1°
*b* = 11.4992 (6) Å	µ = 0.25 mm^−^^1^
*c* = 7.7846 (5) Å	*T* = 296 K
β = 103.287 (6)°	Block, colourless
*V* = 1265.04 (13) Å^3^	0.43 × 0.31 × 0.20 mm
*Z* = 4	

### Data collection

**Table:**

Oxford-Diffraction Gemini S Ultra diffractometer	2253 independent reflections
Radiation source: Enhance (Mo) X-ray Source	1928 reflections with *I* > 2σ(*I*)
graphite	*R*_int_ = 0.018
Detector resolution: 16.0774 pixels mm^-1^	θ_max_ = 25.2°, θ_min_ = 3.2°
ω and φ scans	*h* = −17→16
Absorption correction: multi-scan (*CrysAlis RED*; Oxford Diffraction, 2007)	*k* = −13→13
*T*_min_ = 0.900, *T*_max_ = 0.952	*l* = −7→9
8991 measured reflections	

### Refinement

**Table:**

Refinement on *F*^2^	Primary atom site location: structure-invariant direct methods
Least-squares matrix: full	Secondary atom site location: difference Fourier map
*R*[*F*^2^ > 2σ(*F*^2^)] = 0.031	Hydrogen site location: inferred from neighbouring sites
*wR*(*F*^2^) = 0.085	H-atom parameters constrained
*S* = 1.05	*w* = 1/[σ^2^(*F*_o_^2^) + (0.0422*P*)^2^ + 0.369*P*] where *P* = (*F*_o_^2^ + 2*F*_c_^2^)/3
2253 reflections	(Δ/σ)_max_ = 0.001
163 parameters	Δρ_max_ = 0.27 e Å^−^^3^
0 restraints	Δρ_min_ = −0.24 e Å^−^^3^

### Special details

**Table:**

Experimental. CrysAlis RED, Oxford Diffraction Ltd., Version 1.171.33.32 (release 27-01-2009 CrysAlis171 .NET) (compiled Jan 27 2009,14:17:37) Empirical absorption correction using spherical harmonics, implemented in SCALE3 ABSPACK scaling algorithm.
Geometry. Bond distances, angles *etc*. have been calculated using the rounded fractional coordinates. All su's are estimated from the variances of the (full) variance-covariance matrix. The cell e.s.d.'s are taken into account in the estimation of distances, angles and torsion angles
Refinement. Refinement on *F*^2^ for ALL reflections except those flagged by the user for potential systematic errors. Weighted *R*-factors *wR* and all goodnesses of fit *S* are based on *F*^2^, conventional *R*-factors *R* are based on *F*, with *F* set to zero for negative *F*^2^. The observed criterion of *F*^2^ > σ(*F*^2^) is used only for calculating -*R*-factor-obs *etc*. and is not relevant to the choice of reflections for refinement. *R*-factors based on *F*^2^ are statistically about twice as large as those based on *F*, and *R*-factors based on ALL data will be even larger.

### Fractional atomic coordinates and isotropic or equivalent isotropic displacement parameters (Å^2^)

**Table:**

	*x*	*y*	*z*	*U*_iso_*/*U*_eq_	
S1	1.18567 (3)	0.37673 (4)	0.30521 (6)	0.0398 (1)	
O11	1.21041 (8)	0.26127 (11)	0.26279 (18)	0.0556 (5)	
O12	1.19451 (9)	0.47053 (13)	0.19034 (18)	0.0621 (5)	
N1	1.25128 (9)	0.40557 (11)	0.49616 (19)	0.0418 (4)	
N4	0.78277 (9)	0.36050 (11)	0.36083 (18)	0.0389 (4)	
C1	1.06568 (11)	0.37128 (13)	0.3201 (2)	0.0345 (5)	
C2	1.00490 (12)	0.46245 (14)	0.2581 (2)	0.0420 (5)	
C3	0.91150 (11)	0.45702 (14)	0.2709 (2)	0.0431 (5)	
C4	0.87847 (11)	0.36039 (13)	0.3457 (2)	0.0350 (5)	
C5	0.94078 (11)	0.26993 (14)	0.4104 (2)	0.0383 (5)	
C6	1.03363 (11)	0.27468 (13)	0.3960 (2)	0.0380 (5)	
C41	0.73681 (11)	0.26550 (14)	0.3389 (2)	0.0409 (5)	
C42	0.63915 (11)	0.25305 (14)	0.3574 (2)	0.0405 (5)	
C43	0.58382 (13)	0.34722 (17)	0.3798 (3)	0.0584 (7)	
C44	0.49211 (14)	0.3309 (2)	0.3954 (3)	0.0685 (8)	
C45	0.45425 (13)	0.2225 (2)	0.3881 (3)	0.0638 (8)	
C46	0.50775 (16)	0.1288 (2)	0.3660 (4)	0.0746 (9)	
C47	0.60028 (14)	0.14359 (17)	0.3505 (3)	0.0629 (7)	
H2	1.02730	0.52870	0.20700	0.0500*	
H3	0.86980	0.51960	0.22830	0.0520*	
H5	0.91910	0.20460	0.46480	0.0460*	
H6	1.07550	0.21210	0.43790	0.0450*	
H11	1.23500	0.47150	0.53220	0.0480*	
H12	1.24380	0.35210	0.57000	0.0480*	
H41	0.76760	0.19860	0.30880	0.0490*	
H43	0.60920	0.42360	0.38450	0.0700*	
H44	0.45490	0.39620	0.41160	0.0820*	
H45	0.39080	0.21220	0.39820	0.0760*	
H46	0.48150	0.05290	0.36120	0.0900*	
H47	0.63700	0.07770	0.33510	0.0750*	

### Atomic displacement parameters (Å^2^)

**Table:**

	*U*^11^	*U*^22^	*U*^33^	*U*^12^	*U*^13^	*U*^23^
S1	0.0320 (2)	0.0436 (2)	0.0465 (3)	−0.0013 (2)	0.0148 (2)	−0.0021 (2)
O11	0.0422 (7)	0.0583 (8)	0.0686 (9)	0.0029 (6)	0.0177 (6)	−0.0237 (7)
O12	0.0473 (7)	0.0774 (9)	0.0668 (9)	−0.0024 (6)	0.0241 (6)	0.0241 (7)
N1	0.0351 (7)	0.0369 (7)	0.0540 (9)	−0.0028 (6)	0.0118 (6)	−0.0050 (6)
N4	0.0323 (7)	0.0386 (7)	0.0470 (8)	0.0007 (6)	0.0119 (6)	−0.0024 (6)
C1	0.0308 (8)	0.0361 (8)	0.0373 (8)	−0.0016 (6)	0.0095 (6)	−0.0039 (7)
C2	0.0416 (9)	0.0326 (8)	0.0550 (10)	−0.0002 (7)	0.0176 (8)	0.0056 (7)
C3	0.0383 (9)	0.0348 (8)	0.0579 (11)	0.0064 (7)	0.0143 (8)	0.0051 (8)
C4	0.0320 (8)	0.0354 (8)	0.0385 (8)	−0.0003 (6)	0.0102 (6)	−0.0055 (7)
C5	0.0367 (8)	0.0343 (8)	0.0446 (9)	−0.0019 (6)	0.0109 (7)	0.0041 (7)
C6	0.0336 (8)	0.0347 (8)	0.0443 (9)	0.0027 (6)	0.0064 (7)	0.0031 (7)
C41	0.0359 (9)	0.0387 (9)	0.0488 (9)	0.0015 (7)	0.0112 (7)	−0.0055 (7)
C42	0.0335 (8)	0.0430 (9)	0.0453 (9)	−0.0030 (7)	0.0097 (7)	−0.0031 (7)
C43	0.0378 (10)	0.0477 (10)	0.0915 (15)	−0.0012 (8)	0.0188 (10)	−0.0052 (10)
C44	0.0394 (11)	0.0706 (14)	0.0988 (17)	0.0055 (10)	0.0226 (11)	−0.0102 (13)
C45	0.0366 (10)	0.0868 (16)	0.0701 (14)	−0.0095 (10)	0.0169 (9)	−0.0023 (12)
C46	0.0548 (13)	0.0639 (14)	0.1079 (19)	−0.0227 (11)	0.0245 (13)	−0.0024 (13)
C47	0.0462 (11)	0.0489 (11)	0.0965 (16)	−0.0058 (9)	0.0225 (11)	−0.0086 (11)

### Geometric parameters (Å, °)

**Table:**

S1—O11	1.4337 (13)	C42—C43	1.383 (3)
S1—O12	1.4256 (15)	C43—C44	1.378 (3)
S1—N1	1.6051 (15)	C44—C45	1.358 (3)
S1—C1	1.7737 (17)	C45—C46	1.362 (3)
N4—C4	1.421 (2)	C46—C47	1.387 (3)
N4—C41	1.271 (2)	C2—H2	0.9500
N1—H12	0.8700	C3—H3	0.9500
N1—H11	0.8600	C5—H5	0.9500
C1—C6	1.388 (2)	C6—H6	0.9500
C1—C2	1.384 (2)	C41—H41	0.9500
C2—C3	1.384 (2)	C43—H43	0.9500
C3—C4	1.390 (2)	C44—H44	0.9500
C4—C5	1.395 (2)	C45—H45	0.9500
C5—C6	1.379 (2)	C46—H46	0.9500
C41—C42	1.465 (2)	C47—H47	0.9500
C42—C47	1.375 (3)		
			
S1···H6^i^	3.1100	H2···C5^iii^	2.9900
O11···C6^i^	3.398 (2)	H2···C6^iii^	3.0200
O11···N1^i^	2.9845 (19)	H5···C41	2.7000
O11···H45^ii^	2.6500	H5···H41	2.2600
O11···H6	2.6900	H5···C2^v^	3.0200
O11···H6^i^	2.8400	H5···C3^v^	3.0400
O11···H12^i^	2.1300	H6···O11	2.6900
O12···H2	2.5500	H6···S1^v^	3.1100
O12···H41^iii^	2.6800	H6···O11^v^	2.8400
O12···H47^iii^	2.7900	H11···N4^iv^	2.1400
N1···N4^iv^	2.9955 (18)	H11···C3^iv^	3.0100
N1···O11^v^	2.9845 (19)	H11···C4^iv^	2.8400
N4···N1^iv^	2.9955 (18)	H11···H43^iv^	2.5100
N1···H43^iv^	2.8200	H12···O11^v^	2.1300
N4···H43	2.6700	H41···C5	2.5900
N4···H11^iv^	2.1400	H41···H5	2.2600
C6···O11^v^	3.398 (2)	H41···H47	2.4000
C44···C47^v^	3.542 (3)	H41···O12^vi^	2.6800
C47···C44^i^	3.542 (3)	H43···N4	2.6700
C2···H5^i^	3.0200	H43···H46^vii^	2.5400
C3···H11^iv^	3.0100	H43···N1^iv^	2.8200
C3···H5^i^	3.0400	H43···H11^iv^	2.5100
C4···H11^iv^	2.8400	H45···O11^ix^	2.6500
C5···H2^vi^	2.9900	H46···C43^x^	3.0300
C5···H41	2.5900	H46···H43^x^	2.5400
C6···H2^vi^	3.0200	H46···C46^viii^	2.9600
C41···H5	2.7000	H46···H46^viii^	2.4300
C43···H46^vii^	3.0300	H47···H41	2.4000
C46···H46^viii^	2.9600	H47···O12^vi^	2.7900
H2···O12	2.5500		
			
O11—S1—O12	119.52 (8)	C43—C44—C45	120.8 (2)
O11—S1—N1	106.13 (8)	C44—C45—C46	119.6 (2)
O11—S1—C1	106.51 (7)	C45—C46—C47	120.4 (2)
O12—S1—N1	107.74 (8)	C42—C47—C46	120.39 (19)
O12—S1—C1	107.41 (8)	C1—C2—H2	120.00
N1—S1—C1	109.25 (7)	C3—C2—H2	120.00
C4—N4—C41	118.77 (13)	C2—C3—H3	120.00
H11—N1—H12	109.00	C4—C3—H3	120.00
S1—N1—H11	110.00	C4—C5—H5	120.00
S1—N1—H12	109.00	C6—C5—H5	120.00
S1—C1—C2	120.51 (12)	C1—C6—H6	120.00
S1—C1—C6	119.12 (12)	C5—C6—H6	120.00
C2—C1—C6	120.36 (15)	N4—C41—H41	118.00
C1—C2—C3	119.85 (15)	C42—C41—H41	118.00
C2—C3—C4	120.29 (15)	C42—C43—H43	120.00
C3—C4—C5	119.33 (15)	C44—C43—H43	120.00
N4—C4—C5	122.43 (14)	C43—C44—H44	120.00
N4—C4—C3	118.17 (14)	C45—C44—H44	120.00
C4—C5—C6	120.41 (15)	C44—C45—H45	120.00
C1—C6—C5	119.73 (15)	C46—C45—H45	120.00
N4—C41—C42	124.13 (15)	C45—C46—H46	120.00
C41—C42—C43	122.64 (15)	C47—C46—H46	120.00
C41—C42—C47	118.91 (16)	C42—C47—H47	120.00
C43—C42—C47	118.45 (17)	C46—C47—H47	120.00
C42—C43—C44	120.38 (18)		
			
O11—S1—C1—C2	−141.95 (13)	C2—C3—C4—C5	1.1 (2)
O11—S1—C1—C6	38.53 (15)	N4—C4—C5—C6	−178.65 (14)
O12—S1—C1—C2	−12.78 (15)	C3—C4—C5—C6	−1.8 (2)
O12—S1—C1—C6	167.69 (13)	C4—C5—C6—C1	1.4 (2)
N1—S1—C1—C2	103.82 (13)	N4—C41—C42—C43	8.1 (3)
N1—S1—C1—C6	−75.71 (14)	N4—C41—C42—C47	−172.62 (17)
C41—N4—C4—C3	143.94 (15)	C41—C42—C43—C44	179.57 (18)
C41—N4—C4—C5	−39.2 (2)	C47—C42—C43—C44	0.3 (3)
C4—N4—C41—C42	177.62 (14)	C41—C42—C47—C46	−179.4 (2)
S1—C1—C2—C3	−179.89 (12)	C43—C42—C47—C46	−0.1 (3)
C6—C1—C2—C3	−0.4 (2)	C42—C43—C44—C45	−0.4 (3)
S1—C1—C6—C5	179.21 (12)	C43—C44—C45—C46	0.3 (4)
C2—C1—C6—C5	−0.3 (2)	C44—C45—C46—C47	−0.2 (4)
C1—C2—C3—C4	0.0 (2)	C45—C46—C47—C42	0.1 (4)
C2—C3—C4—N4	178.08 (14)		

Symmetry codes: (i) *x*, −*y*+1/2, *z*−1/2; (ii) *x*+1, *y*, *z*; (iii) −*x*+2, *y*+1/2, −*z*+1/2; (iv) −*x*+2, −*y*+1, −*z*+1; (v) *x*, −*y*+1/2, *z*+1/2; (vi) −*x*+2, *y*−1/2, −*z*+1/2; (vii) −*x*+1, *y*+1/2, −*z*+1/2; (viii) −*x*+1, −*y*, −*z*+1; (ix) *x*−1, *y*, *z*; (x) −*x*+1, *y*−1/2, −*z*+1/2.

### Hydrogen-bond geometry (Å, °)

**Table:**

*D*—H···*A*	*D*—H	H···*A*	*D*···*A*	*D*—H···*A*
N1—H11···N4^iv^	0.86	2.14	2.9955 (18)	171
N1—H12···O11^v^	0.87	2.13	2.9845 (19)	171

Symmetry codes: (iv) −*x*+2, −*y*+1, −*z*+1; (v) *x*, −*y*+1/2, *z*+1/2.
